# A Cluster of Lymphocytic Choriomeningitis Virus Infections Transmitted Through Organ Transplantation — Iowa, 2013

**Published:** 2014-03-21

**Authors:** Ilana J. Schafer, Rachel Miller, Ute Ströher, Barbara Knust, Stuart T. Nichol, Pierre E. Rollin

**Affiliations:** 1 EIS officer, CDC; 2 University of Iowa Carver College of Medicine; 3 Viral Special Pathogens Branch, National Center for Emerging and Zoonotic Infections, CDC

On April 26, 2013, the United Network for Organ Sharing reported to CDC a cluster of ill organ transplant recipients in Iowa with a common organ donor. Infection with lymphocytic choriomeningitis virus (LCMV) was suspected. LCMV is a rodent-borne virus that most commonly causes nonfatal, influenza-like illness and occasional aseptic meningitis, but when transmitted through organ transplantation or in utero can cause severe, life-threatening disease.

The organ donor, a man aged 49 years, had experienced a headache and vomiting and was then found unresponsive in his home on March 23, 2013. Imaging revealed a large intracerebral hemorrhage, and he was declared brain dead on March 24. Four patients received donated organs or tissues on March 26, and three were hospitalized between April 12 and 16 with symptoms including fever, abdominal pain, diarrhea, altered mental status, and respiratory compromise. At the time of CDC’s notification, patient A (liver recipient) and patient C (left kidney recipient) were hospitalized in critical condition and patient B (right kidney recipient) had been discharged with resolving symptoms. Patient D (cornea recipient) was asymptomatic.

Diagnostic testing at CDC confirmed LCMV as the causative agent. LCMV was detected in liver and/or blood samples from patients A and B and in donor aortic endothelial cells by reverse transcription–polymerase chain reaction. All three ill recipients developed virus-specific immunoglobulin M. Patient D tested negative for LCMV.

Immunosuppression was reduced in all three ill recipients. Treatment with oral ribavirin was commenced for patient B on May 2. Patients A and C were started on intravenous ribavirin treatment on May 3, with patient C having received oral ribavirin for 2 days prior. Patients A and C were also treated with intravenous immunoglobulin.

All nontransplanted donor organs and tissues were traced and destroyed or sent to CDC for testing, including plasma that was donated 2 days before death. No definitive evidence of rodent exposure was discovered for the donor, although he had spent much time outside along the Mississippi River.

Patient A died on May 11. As of February 20, 2014, patient B had recovered except for mild memory deficits. Patient C was in a nursing facility in fair condition, with ongoing memory deficits and with allograft failure necessitating return to dialysis.

This reported cluster is the fifth LCMV organ transplant-associated cluster documented in the United States, with 14 LCMV-infected organ recipients, including 11 deaths, previously described (1–3). All five clusters have occurred in the past decade. The three previous cornea recipients also did not develop LCMV infections (1,2). Physicians and public health practitioners should be aware that organ donors with suspected central nervous system infection, and some with intracranial hemorrhage without evidence of infection, could be infected with LCMV, especially when rodent exposure has occurred. Testing for LCMV should be considered in organ recipients who develop febrile illness, neurologic changes, or multiorgan dysfunction in the early posttransplant period, especially if multiple recipients from the same donor become ill. The recommended treatment for LCMV infections obtained through organ transplantation includes reduced immunosuppression and ribavirin. The efficacy of ribavirin in these cases has not been determined; however, early detection of LCMV and prompt treatment initiation might improve outcome.
